# Identification and Expression Analysis of the β-1,3-Glucanase Gene Family in the Mycoparasitic *Alternaria alternata* KMR13 from Rose Powdery Mildew

**DOI:** 10.3390/microorganisms14061298

**Published:** 2026-06-09

**Authors:** Yanping Tang, Ruotian Gao, Chen Chen, Mengling Yan, Jing Li

**Affiliations:** College of Biological Science and Food Engineering, Forest Resources Exploitation and Utilization Engineering Research Center for Grand Health of Yunnan Provincial Universities, Southwest Forestry University, Kuming 650224, China; typ1872739499@163.com (Y.T.); gruotian@163.com (R.G.); 15808739473@163.com (C.C.); 18887478794@163.com (M.Y.)

**Keywords:** mycoparasitic, cell wall degrading enzymes, β-1,3-glucanase, bioinformatics, prokaryotic expression

## Abstract

The β-1,3-glucanase gene family plays an important role in fungal cell wall degradation and is closely associated with the mycoparasitic interactions of biocontrol fungi. In this study, the β-1,3-glucanase gene family was identified in the mycoparasitic *Alternaria alternata* strain KMR13, and its expression patterns under *Podosphaera pannosa* spore induction were analyzed to investigate the role of the gene *Aag25* in the degradation of *P. pannosa* spores. The β-1,3-glucanase gene was extracted from the genome using the bioinformatics method. According to the expression level of spore-induced genes of *P. pannosa*, the GH17 family genes that may be involved in mycoparasitism were screened, cloned, and expressed. The expressed protein was purified, and its activity and ability to destroy spores of *P. pannosa* were determined. A total of 30 β-1,3-glucanase genes were identified in strain KMR13, including 18 members of the GH16 family, 8 of the GH17 family, and 2 genes each from the GH64 and GH81 families. Transcriptome analysis revealed that 23 of these genes were expressed under spore induction. Based on differential expression analysis, the β-1,3-glucanase gene *Aag25* was selected for prokaryotic expression. The recombinant Aag25 protein (~32.08 kDa) was successfully induced and purified, exhibiting an enzymatic activity of 1.464 U/mg protein. Functional assays demonstrated that recombinant Aag25 could effectively disrupt the cell walls of *P. pannosa* spores, leading to spore rupture and cytoplasmic leakage. These results indicate that Aag25 plays an important role in fungal cell wall degradation during the mycoparasitic process of strain KMR13 and provides a potential enzymatic resource for the development of biological control strategies targeting fungal pathogens.

## 1. Introduction

The Chinese rose (*Rosa chinensis* Jacq.), a perennial shrub belonging to the genus *Rosa* in the family Rosaceae, is known as the “Queen of Flowers” [1]. The Chinese rose possesses both ornamental and medicinal value and holds a significant position in the floral industry [2]. Powdery mildew, caused by the obligate biotrophic fungus *Podosphaera pannosa*, has become a major obstacle to the global cut-flower industry for Chinese roses [3,4,5], posing a serious threat to their growth, development, and economic viability [6].

Currently, research on the mechanisms of mycoparasitism primarily focuses on cell wall–degrading enzymes and antifungal secondary metabolites [7]. Mycoparasitic can produce enzymes that degrade the cell walls of host fungi; the interaction between mycoparasites and host fungi generates antimicrobial secondary metabolites, namely toxins, which can kill the host fungi. Following toxin exposure, the growth of the pathogenic fungi is inhibited, but their cell walls remain intact. Instead, the cells undergo deformation, and their contents become concentrated and leak out, leading to cell death [8,9]. The fungal cell wall is primarily composed of polysaccharides (approximately 80%) and proteins (3–20%), with relatively small amounts of lipids, pigments, and inorganic salts. In filamentous fungi, the cell wall structure typically consists of glucans, glycoproteins, proteins, and chitin arranged in layers. Structural polysaccharides such as β-glucans, chitin (or chitosan in some fungi), and cellulose microfibrils form the structural framework that provides rigidity and shape to the fungal cell wall [10]. The fungal cell wall is essential for maintaining cellular integrity; its degradation disrupts osmotic balance, leading to increased internal turgor pressure and eventual cell lysis. Therefore, targeting cell wall synthesis may serve as a promising antifungal strategy [11]. Therefore, investigating hydrolases such as chitinases, glucanases, and proteases, which degrade fungal cell walls, is crucial for understanding the mechanisms underlying mycoparasitic interactions.

β-1,3-glucan is a major structural component of fungal cell walls and represents a class of high molecular weight polysaccharides widely distributed in nature. Its backbone consists of glucose units linked by β-1,3-glycosidic bonds. β-1,3-glucanases are enzymes capable of hydrolyzing β-1,3-glycosidic linkages and play important roles in the degradation, remodeling, and biological utilization of β-1,3-glucans [12]. These enzymes are widely distributed in bacteria, fungi, algae, certain animals, and higher plants, with fungi representing one of the major sources [13]. β-1,3-glucanases are important antifungal enzymes secreted by biocontrol microorganisms, and plants can also induce the production of β-1,3-glucanases as part of their defense response against fungal pathogens [14]. By degrading β-1,3-glucan chains in fungal cell walls, β-1,3-glucanases can cause hyphal breakage or deformation, resulting in cytoplasmic leakage and inhibition of spore germination. Nicolas et al. [15] first elucidated the role of GH55 family glucanases in fungal conidial development, demonstrating that β-1,3-glucanases participate in fungal cell wall hydrolysis; specifically, exo-β-1,3-glucanases play an important role in the maturation of conidia in *Aspergillus fumigatus*.

In this study, the β-1,3-glucanase gene was extracted from the genome of strain KMR13 via the bioinformatics method. Combined with transcriptomic data induced by *P. pannosa* spores, genes encoding cell wall-degrading enzymes with high expression levels or significant differential expression were screened for validation using RT-qPCR. Subsequently, key genes were selected for prokaryotic expression and enzymatic activity assays. This work aims to provide a theoretical basis for elucidating the mycoparasitic biocontrol mechanism of strain KMR13 and facilitate its further development and application as a biological control agent.

## 2. Materials and Methods

### 2.1. Materials

The test strain was *Alternaria alternata* KMR13 [15] isolated from *P. pannosa*. The strain is preserved in the Department of Biochemistry of the School of Biological and Food Engineering at Southwest Forestry University.

The following culture media were employed in this study: potato dextrose agar (PDA), potato dextrose broth (PDB), Luria–Bertani (LB) solid medium, and LB liquid medium. The PDA was prepared by infusing 200 g of sliced potato in boiling distilled water, followed by the addition of 20 g of glucose and 20 g of agar, with the final volume adjusted to 1000 mL using sterile water. The pH was not modified. The PDB was formulated identically, with the omission of the agar. The LB solid medium consisted of 10 g of tryptone, 10 g of sodium chloride, 5 g of yeast extract, and 15 g of agar, brought to a final volume of 1000 mL with water. The LB liquid medium was prepared according to the same composition, except for the exclusion of the agar. All reagents used in this experiment were purchased from Sangon Biotech (Shanghai) Co., Ltd. (Shanghai, China), and the instruments and equipment were provided by the Biochemistry and Molecular Biology Laboratory, College of Biology and Food Engineering, Southwest Forestry University (Kunming, China).

### 2.2. Methods

#### 2.2.1. Identification and Characterization of β-1,3-Glucanase Genes

Following the methods for identifying and classifying β-1,3-glucanase genes described by Wang et al. [16] and Sui Wenjing [17], we screened for members of the β-1,3-glucanase gene family based on existing genome annotation results. We utilized the Pfam database (http://pfam.xfam.org/ accessed on 20 February 2026) to screen for GH16, GH17, GH64, GH81 glycosidase family genes [18]. We used the online tool Conserved Domain Database (CDD) (Available online: http://www.ncbi.nlm.nih.gov/cdd/ accessed on 20 February 2026) [19] and SMART [20] (Available online: http://smart.embl-heidelberg.de/ accessed on 20 February 2026) to predict domains in the screened β-1,3-glucanase genes. Duplicate and missing domain sequences were removed, and the intersection of the two sets was calculated to ultimately identify candidate genes for the β-1,3-glucanase gene family.

#### 2.2.2. Bioinformatics Analysis of β-1,3-Glucanase Genes

The physicochemical properties of the encoded proteins from the screened *Aags* gene sequences were predicted online using ProtParm (https://www.psort.org/psortb/ accessed on 27 April 2026) [21]; signal peptide sites were predicted using SignalP 5.0 (https://services.healthtech.dtu.dk/services/SignalP-5.0/ accessed on 20 February 2026) [22]; TMHMM (https://services.healthtech.dtu.dk/services/TMHMM-2.0/ accessed on 20 February 2026) was used to predict transmembrane structures [23]; and WoLF PSORT (https://wolfpsort.hgc.jp/ accessed on 20 February 2026) was used to predict subcellular localization [24].

Based on the genome file and annotation information file, TBtools [25] software (v2.472) was used to visually analyze the results of *Aags* gene structure; the protein motifs of *Aags* were analyzed using the online MEME database, with the maximum number of motifs set to 10 and other parameters left at their default values [26]; the results of the protein motif analysis were visualized using TBtools software; chromosomal mapping of *Aags* was performed using TBtools software; cis-acting elements in the promoter region were predicted using the online prediction website Plantcare (http://bioinformatics.psb.ugent.be/webtools/plantcare/html/ accessed on 20 February 2026) [27]; and the results were visualized using TBtools software.

#### 2.2.3. Analysis of Target Gene Expression Induced by Spores of *Podosphaera pannosa*

After harvesting the spore, the suspension was subjected to repeated freeze-thaw cycles at −20 °C, followed by mechanical disruption using a pre-chilled mortar and pestle. The ground spores were collected and examined under a microscope. The spores were then sterilized at 121 °C for 20 min, and the resulting spore walls were used as the inducer. After activating strain KMR13 on the PDA medium, second-generation mycelium was inoculated into 100 mL of PDB liquid medium and cultured for 24 h at 28 °C on a constant-temperature shaker at 150 r/min. The mycelium was then induced with 2.5 g/L of spores at 0, 24, 48, 72, and 96 h, with three biological replicates per treatment group. Upon completion of induction, the induced mycelia were removed under sterile conditions, flash-frozen in liquid nitrogen, and stored. They were then sent to Hangzhou Lianchuan Biotechnology Co., Ltd. for transcriptomic sequencing. The expression levels of the β-1,3-glucanase GH16, GH17, GH64, and GH81 gene families were determined for both the experimental and control groups, and the data were analyzed using TBtools to generate heat maps.

RNA was extracted using the TianGen RNA Extraction Kit (Tiangen Biotech (Beijing) Co., Ltd., Beijing, China), and the first strand of cDNA was synthesized using a reverse transcription kit in a 20 μL reaction system. Target genes with high expression levels or significant expression differences were selected for RT-qPCR. Here, cDNA was used as the template, and actin is an internal reference gene. Primers were designed using primer Primer 5.0 software. The sequences of the internal control and gene-specific primers are shown in Table 1. Relative quantification was calculated using the 2^−ΔΔCt^ method, and data were analyzed using one-way ANOVA with SPSS 26.0 software.

#### 2.2.4. Construction of the pET22b-Aag25 Expression Vector

Using the cDNA sequence of the *Aag25* β-1,3-glucanase gene from strain KMR13 as a template, the signal peptide at the N-terminus of the *Aag25* gene was removed. The target gene sequence, containing the conserved and functional domains, was then synthesized via chemical synthesis by Shanghai Genomics. The resulting protein sequence was ligated to the expression vector pET-22b(+) using dual-restriction enzyme digestion with *NdeI* and *XhoI*, and the ligation was verified via dual-restriction enzyme digestion to obtain the recombinant plasmid pET22b-*Aag25*. The recombinant plasmid was transformed into *E. coli* Rosetta (DE3) competent cells, heat-shocked at 42 °C, and then spread onto plates containing 50 µg/mL of ampicillin for incubation at 37 °C. Single-colony pickings were selected for PCR verification. Simultaneously, the plasmid was extracted using the SanPrep Column-Based Plasmid Extraction Kit (Shanghai, China, GenScript) and sent to GenScript Biotech Co., Ltd. in Shanghai for unidirectional sequencing of the target fragment. After verifying that the sequencing results were correct, the recombinant strain pET22b(+)-*Aag25/E. coli* Rosetta (DE3) was obtained.

#### 2.2.5. Prokaryotic Expression and Protein Purification of Aag25

Recombinant strain pET22b(+)*-Aag25/E. coli* Rosetta (DE3) was streaked onto LB plates containing 50 µg/mL of ampicillin and incubated at 37 °C for 16 h. Then, 3–5 single-colony colonies were transferred to antibiotic-containing liquid medium and incubated at 37 °C for 16 h. When the OD_600_ = 0.6, we added 0.5 mM of IPTG as an inducer and continue cultivation, followed by incubation overnight at 20 °C and for 6 h at 37 °C, respectively, using a sample without the inducer as a negative control. After incubation, we transferred the bacterial culture to 50 mL centrifuge tubes, centrifuged them at 4000 rpm for 10 min using a refrigerated centrifuge, discarded the supernatant, and collected the cell pellet. We resuspended the collected cell pellet in Buffer A and used an ultrasonicator to ensure complete dissolution (power = 140 W, 3 s on, 5 s off, total duration = 30 min). They were then centrifuged at 12,000 rpm and 4 °C for 20 min to collect the supernatant (i.e., crude enzyme solution) and the pellet. We dissolved the pellet in Buffer B and prepared samples from both the supernatant and the pellet proteins for gel electrophoresis. Next, we cultured the bacterial suspension in medium containing the appropriate antibiotic. When the OD value reached 0.6, we added 0.5 mM of IPTG as an inducer and incubated it overnight at 20 °C for high-yield expression. Finally, we centrifuged it to collect the bacterial cells.

#### 2.2.6. Detection of the Aag25 Protein

The molecular weight of the recombinant Aag25 protein was determined using SDS-PAGE. The purified protein was further validated using western blot analysis. A 5% concentrating gel and a 12% separating gel were used. For the western blot, the primary and secondary antibodies were anti-His mouse monoclonal antibody (Shanghai Bio-Engineering, Shanghai, China) and goat anti-mouse antibody (Shanghai Bio-Engineering), respectively, and validation was performed using a tag-specific antibody.

#### 2.2.7. Determination of Enzyme Activity

(1) Preparation of the Glucose Standard Curve

We prepared a 10 mg/mL glucose standard solution. Using the volumes listed in Table 2, we prepared glucose solutions of various concentrations and added 35 μL of Reagent 1 to each, mixed them thoroughly, and placed them in a 37 °C water bath for 60 min. We then added 230 μL of Reagent 2 to each, mixed them thoroughly, and placed them in a boiling water bath for 5 min (wrapping the well openings with plastic wrap to prevent the lids from popping off). They were cooled under running water before transferring 200 μL to a 96-well plate, and we recorded the absorbance of each well at 540 nm. Using the concentration of the standard solution (x, mg/mL) and the absorbance ΔA(standard) (y, ΔA(standard)), we constructed a standard curve. Lastly, we substituted ΔA(measured) (y, ΔA(measured)) into the formula to calculate the x value (mg/mL), representing the reducing sugar content in the sample.

(2) Enzyme Activity Assay

The protein concentration of the sample was 0.5 mg/mL, and the sample was diluted 10-fold. For the preparation of inactivated samples, 35 µL of the sample supernatant was transferred to a 1.5 mL Eppendorf tube and placed in a boiling water bath for 5 min, with the tube wrapped in plastic film to prevent the cap from popping off. The inactivated sample was then used as a control tube for the assay, and the reagents were added to a 1.5 mL Eppendorf tube in the order listed in Table 3.

It was then mixed thoroughly, placed in a boiling water bath for 5 min (wrapping the top with plastic wrap to prevent the lid from popping off) and cooled under running water before we transferred 200 μL to a 96-well plate and recorded the absorbance of each well at 540 nm. We labeled the results as A(test), A(control), A(standard), and A(blank), respectively. We calculated ΔA(test) = A(test) − A(control) and ΔA(standard) = A(standard) − A(blank). One enzyme activity unit is defined as the production of 1 mg of reducing sugar per hour per milligram of tissue protein:

U = X ÷ Cpr × F(1)

where U is the enzyme activity (U/mg protein); Cpr is the sample protein concentration (mg/mL); and F is the sample dilution factor.

#### 2.2.8. Damage to Spores of *Podosphaera pannosa*

We added 100 µL of the Aag25 enzyme solution obtained after renaturation to a sterile 1.5 mL centrifuge tube and added 0.01 g of *P. pannosa* spores to each tube to ensure the spores were evenly distributed in the enzyme solution. We then incubated them at 25 °C for 1, 2, 4, 6, and 8 days, respectively. Next, we used 100 µL of sterile water to soak the *P. pannosa* spores as a control. Then, we performed three biological replicates for each group. After sampling, we stain them with 0.1% trypan blue for 5 min and observed the destruction of the *P. pannosa* spores under a light microscope.

## 3. Results

### 3.1. Basic Physicochemical Properties, Signal Peptide, and Subcellular Localization Predictions

The screening identified a total of 30 β-1,3-glucanase genes within the genome of *Alternaria alternata* KMR13 (Table 4), comprising 18 members of the GH16 family, 8 members of the GH17 family, 2 members of the GH64 family, and 2 members of the GH81 family. These genes were designated *Aag1*–*30* based on their sequential chromosomal locations. The encoded proteins ranged in length from 270 to 1807 amino acids, with corresponding molecular weights ranging from 29.17 to 203.69 kDa and predicted isoelectric points (pI) between 4.20 and 8.84. With the exceptions of *Aag11*, *Aag14*, and *Aag28*, which were predicted to be basic proteins, the majority of the identified proteins exhibited acidic pI values. Signal peptide prediction revealed the presence of a putative N-terminal secretory signal peptide in 16 of the deduced amino acid sequences, whereas no signal peptide was predicted for the remaining 14 genes. Subcellular localization analysis indicated that the majority of the proteins were destined for the extracellular space (18), with smaller subsets predicted to localize to the plasma membrane (5), mitochondria (4), and peroxisomes (2). *Aag1* alone was predicted to reside in the cytoplasm. Transmembrane helix prediction identified seven proteins (*Aag10*, *Aag11*, *Aag12*, *Aag13*, *Aag14*, *Aag16*, and *Aag20*) as possessing one or more transmembrane domains, while the remainder were classified as non-transmembrane proteins.

### 3.2. Gene Structure and Protein Motif Analysis

**Figure 1:**
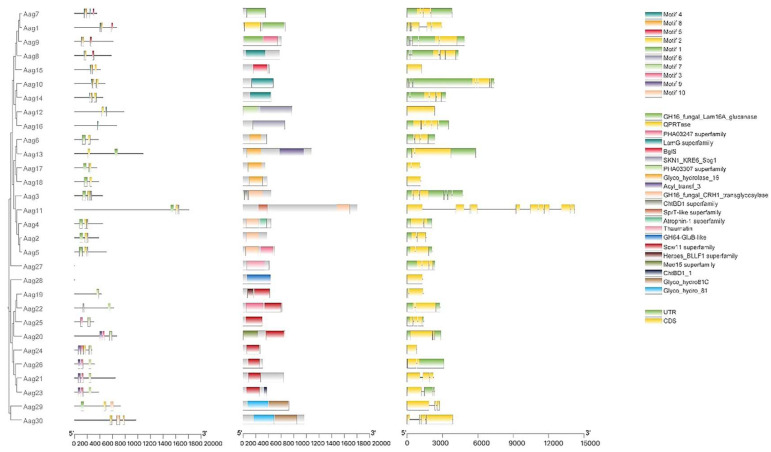
Protein motif, conserved domain, and gene structure prediction of *Aags* gene.

The results for the *Aags* gene domain prediction are shown in Figure 1. All 18 GH16 gene family members contained the glycosidase 16 family domain, as well as 10 other specific domains. Among these, the LamG superfamily domain was present only in *Aag8*, *Aag10*, and *Aag14*, the SKN1_KRE6_Sbg1 domain was present only in *Aag12* and *Aag16*, and the BglS domain was present only in *Aag15*. All eight GH17 gene families contained the glycosidase 17 family domain, as well as four unique domains, among which the Herpes_BLLF1 superfamily domain was present only in *Aag9* and the Med15 superfamily domain was present only in *Aag20*. Both GH64 gene families contained the glycosidase 64 family domain. Both GH81 gene families possessed the glycosidase 81 family domain, indicating that the β-1,3-glucanase gene families in KMR13 are structurally diverse. Analysis of the conserved motifs in β-1,3-glucanase family proteins revealed 10 conserved motifs in the β-1,3-glucanase protein sequences. The distribution of motifs among the β-1,3-glucanase proteins within the same branch was similar; the GH16 family primarily contained Motif 1, Motif 2, Motif 4, and Motif 6, and the GH17 family primarily contained Motif 3, Motif 7, and Motif 9. Meanwhile, the GH64 family lacked Motifs 1–10, and the GH81 family primarily contained Motif 2 and Motif 10. *Aag2*, *Aag3*, *Aag4*, *Aag5*, and *Aag11* shared consistent motifs, while *Aag20*, *Aag21*, *Aag23*, and *Aag26* shared consistent motifs, suggesting that they possess similar structures and functions. However, *Aag16* contained only Motif 4, and *Aag19* contained only Motif 7, suggesting that these motifs play important roles in these genes. Gene structural analysis indicated that members of the KMRChis gene family contained 1–6 introns and 1–10 exons. Among them, *Aag11* contained the highest number of exons (10), while *Aag9* contained the highest number of introns (six). The UTR distribution pattern shows that a total of 10 *Aags* genes lacked UTR regions, specifically *Aag11*, *Aag12*, *Aag15*, *Aag17*, *Aag18*, *Aag21*, *Aag24*, *Aag28*, *Aag29*, and *Aag30*.

### 3.3. Chromosomal Localization and Prediction of Promoter Cis-Acting Elements

**Figure 2:**
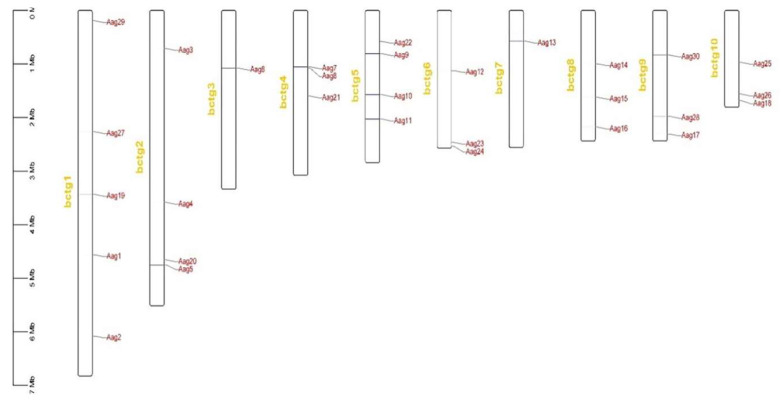
Distribution of *Aags* gene on chromosome.

The results of chromosomal localization of the β-1,3-glucanase genes in strain KMR13 are shown in Figure 2. In this strain, the β-1,3-glucanase genes were unevenly distributed across 10 chromosomes, with no tandem repeats observed. Specifically, *Aag1*, *Aag2*, *Aag19*, *Aag27*, and *Aag29* were located on the same chromosome, while *Aag9*, *Aag10*, *Aag11*, and *Aag22* were located on another chromosome; these two chromosomes contained a relatively large number of genes. Chromosomes Bctg3 and Bctg7 had the fewest genes, each containing only one gene.

**Figure 3:**
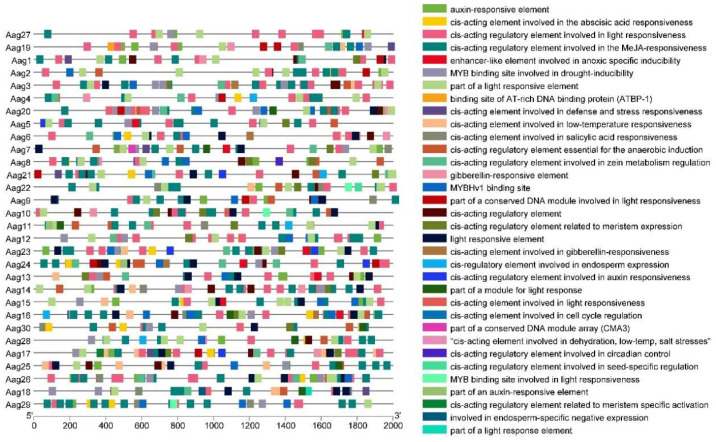
Prediction of cis-acting elements of *Aags* gene promoter.

The results of the cis-regulatory element prediction for *Aags* gene promoters are shown in Figure 3. Among the 30 *Aags* genes, a total of 34 cis-regulatory elements were identified, including hormone-responsive, light-responsive, and circadian rhythm-related elements, as well as stress-responsive elements. Overall, hormone response and light or circadian rhythm-related elements predominated, followed by stress response, general regulation, development- or tissue-specific, and hypoxia or anaerobic induction elements. Among these, *Aag26* possessed the highest number of cis-regulatory elements, while *Aag27* possessed the fewest. MeJA-responsive elements were distributed across the promoters of all 30 *Aags* genes, suggesting that this family is likely involved in jasmonic acid-related defense and stress regulation pathways. ABA response elements were also detected in all 30 genes, suggesting that the family as a whole is closely associated with ABA-mediated drought and osmotic stress signaling. Light response elements were also detected in all 30 genes, with the highest number found in *Aag26*, indicating that it may be more susceptible to changes in light intensity or photoperiod. Circadian rhythm-related elements were detected only in *Aag8*, suggesting that circadian regulation may be a specific feature of this gene.

### 3.4. Analysis of Target Gene Expression Induced by Spores of Podosphaera pannosa

**Figure 4:**
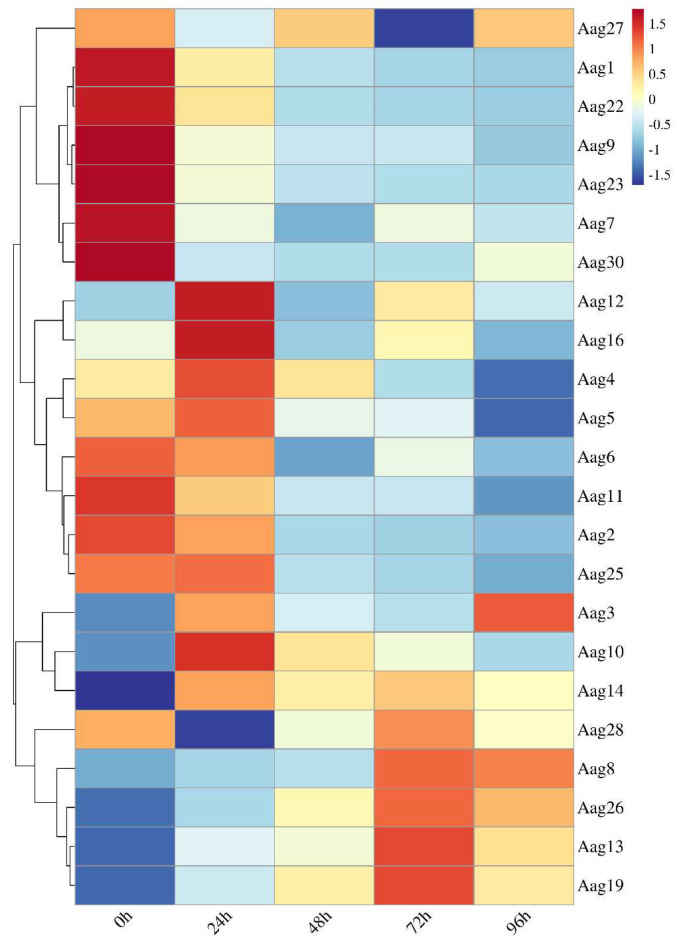
Expression of *Aags* gene induced by spores.

The expression levels of the β-1,3-glucanase genes are shown in Figure 4. Of the 30 β-1,3-glucanase genes in strain KMR13, 23 were detected as expressed in the transcriptome, while *Aag15*, *Aag17*, *Aag18*, *Aag20*, *Aag21*, *Aag24*, and *Aag29* were not. *Aag25* exhibited the highest expression level, with an FPKM value as high as 436.09 at 24 h post induction. *Aag10* followed, reaching a peak FPKM of 229.92 at 24 h post induction. Following induction with rose powdery mildew spores (a suspension of whole spores inactivated by high-temperature treatment), the β-1,3-glucanase genes exhibited differential expression between 0 and 96 h post induction. Among them, *Aag3*, *Aag8*, *Aag10*, *Aag13*, *Aag14*, *Aag19*, and *Aag26*—a total of seven genes—were upregulated within the 0–96-h window. These genes may play a role during pathogen infection. A total of 10 genes showed sustained downregulation throughout the induction process. The reason for their continued downregulation despite the influence of the inducer warrants further investigation. *Aag4*, *Aag5*, and *Aag25* showed upregulated expression between 0 and 24 h, followed by a significant downregulation between 48 and 96 h, indicating a trend of induction-mediated expression changes. These four genes may play a regulatory role during the early stages of infection.

**Figure 5:**
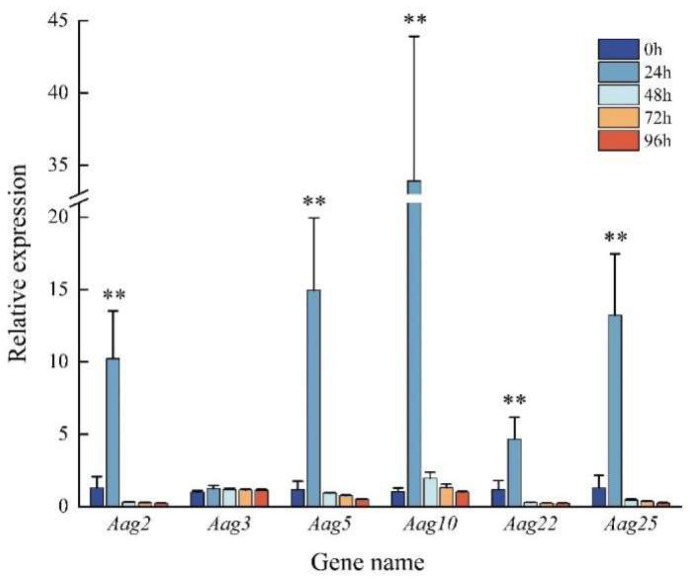
Relative expression of *Aags* gene induced by spore wall of *Podosphaera pannosa*. ** indicates a significant difference at *p* < 0.01.

Six genes with high expression levels or large fold changes were selected for RT-qPCR validation. The expression patterns of these six *Aags* genes were generally consistent with the transcriptomic results. As shown in Figure 5, with the exception of *Aag3*, the expression of nearly all *Aags* genes was significantly upregulated 24 h after spore induction, reaching peak levels at 24 h and exhibiting an induced expression pattern. All six *Aags* genes showed significant upregulation 24 h after induction, with *Aag5* and *Aag10* increasing 12.66-fold and 32.68-fold, respectively, indicating that spores induce the expression of β-1,3-glucanase genes.

### 3.5. Construction of the Recombinant Aag25 Protein Expression Vector

**Figure 6:**
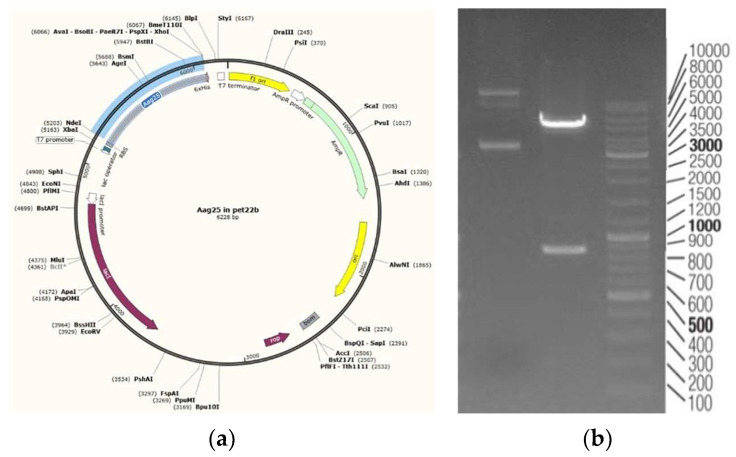
Construction result of recombinant plasmid. (**a**) Vector information. (**b**) Restriction enzyme digestion map of the recombinant plasmid.

The chemically synthesized recombinant plasmid pET22b(+)-Aag25 was transformed into competent *E. coli* Rosetta (DE3) cells using the freeze-thaw method. Following antibiotic selection, the plasmids from positive transformants were double-digested with *NdeI* and *XhoI* restriction enzymes. The results are shown in Figure 6. Following digestion with NdeI and XhoI, electrophoresis revealed approximately two bands with sizes of 861 bp and 5367 bp. This indicates that the construction of the recombinant Aag25 expression vector pET22b(+)-*Aag25/E. coli* Rosetta (DE3) was successful.

### 3.6. Expression and Purification of the Recombinant Aag25 Protein

**Figure 7:**
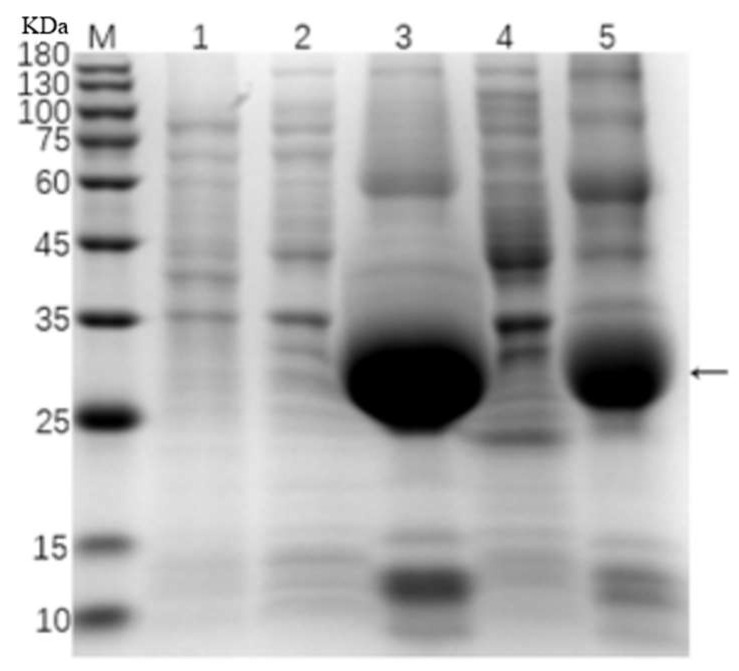
SDS-PAGE analysis of fusion protein expression. (M) Protein marker. The arrow indicates the target protein band. (1) Total protein before induction. (2) Supernatant at 20 °C. (3) Precipitate at 20 °C. (4) Supernatant at 37 °C. (5) Precipitate at 37 °C.

SDS-PAGE electrophoresis was performed on the total recombinant bacterial protein, the supernatant after cell lysis, and the pellet, as shown in Figure 7. Following 6 h of IPTG induction at 20 °C and 37 °C, a distinct dark band between 25 kDa and 35 kDa was observed in the cell lysis pellet, consistent with the protein’s molecular weight of 32.08 kDa. This indicates that the Aag25 gene was successfully cloned and expressed in *E. coli* Rosetta (DE3) and that the highest yield of Aag25 protein was produced under induction conditions at 20 °C, with the protein primarily distributed in the pellet.

**Figure 8:**
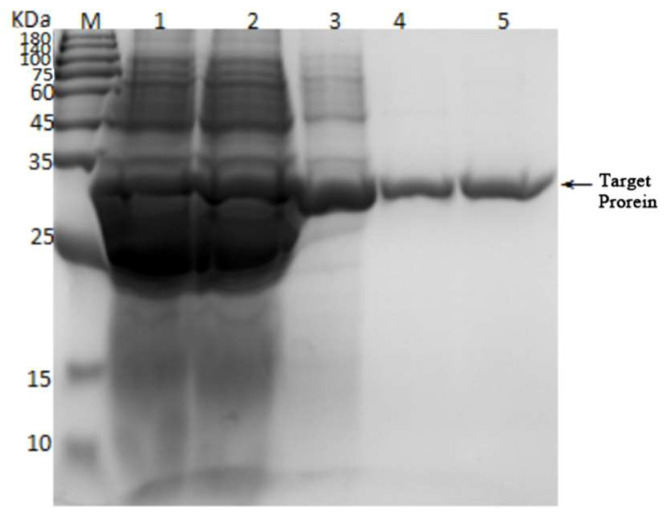
Plot of SDS-PAGE analysis of fusion protein nickel-agarose affinity chromatography. (M) Protein marker. (1) Sample. (2) Elution. (3) 20 mM imidazole elution fraction. (4) 50 mM imidazole elution fraction. (5) 500 mM imidazole elution fraction.

The results of nickel-agarose affinity chromatography of the fusion protein are shown in Figure 8. A distinct single band appeared in all three elution fractions, with a molecular weight ranging from 25 kDa to 35 kDa. However, the target protein band was more pronounced in Fractions 4 and 5, and no other bands were visible to the naked eye. This indicates that the purification was successful. Fractions 4 and 5, which yielded the best results, can be combined for subsequent SDS-PAGE analysis and western blot validation.

### 3.7. Validation of the Aag25 Protein

**Figure 9:**
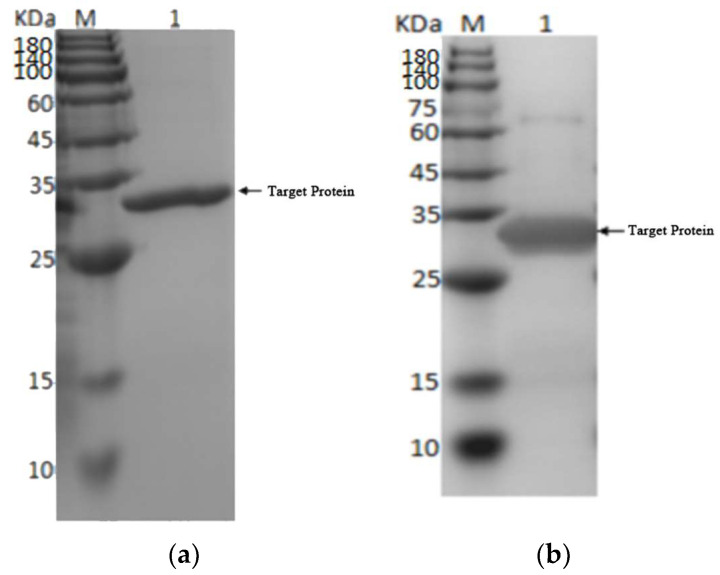
Final purified protein result. (**a**) SDS-PAGE analysis of the final purified protein. (**b**) Western blot analysis of the final purified protein.

To further confirm that the purified protein was the target protein, the purified recombinant protein was subjected to SDS-PAGE analysis and western blot validation. As shown in Figure 9, after purification, SDS-PAGE analysis revealed a distinct band at a position close to the theoretical molecular weight, indicating that the fusion protein had been successfully purified. The western blot results showed a distinct band at the corresponding position, confirming that the protein was the target protein and that its size matched the expected molecular weight. Taken together, these results confirm that the target protein was expressed, was insoluble, and was successfully purified.

### 3.8. Enzymatic Activity of the Aag25 Protein and Its Destructive Effect on Spores of Podosphaera pannosa

#### 3.8.1. Calibration Curve and Analysis of Aag25 Enzymatic Activity

**Figure 10:**
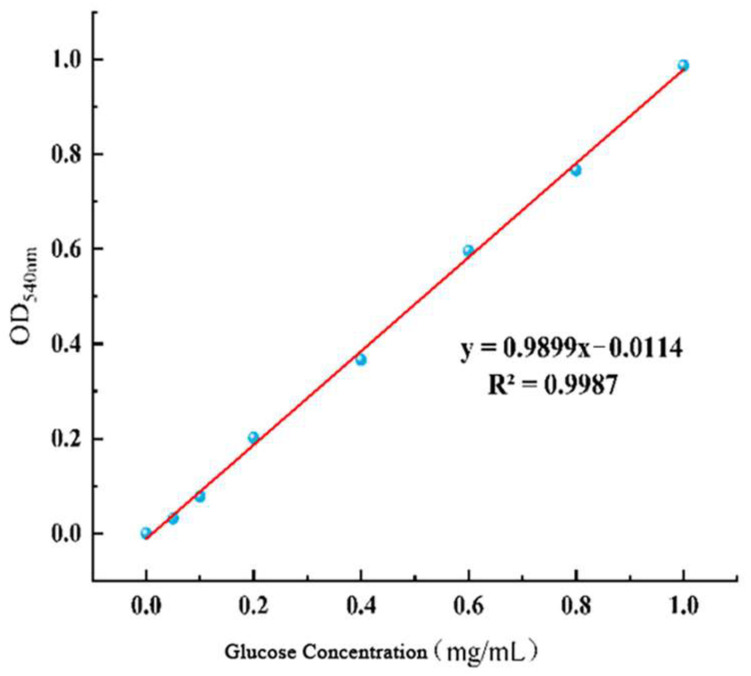
Glucose standard curve.

Figure 10 shows the glucose standard curve, with an R^2^ value of 0.9987 and the equation y = 0.9899x − 0.0114. Based on Equation (1), the enzymatic activity of Aag25 was determined to be 1.464 U/mg protein.

#### 3.8.2. Damage to Spores of *Podosphaera pannosa*

**Figure 11:**
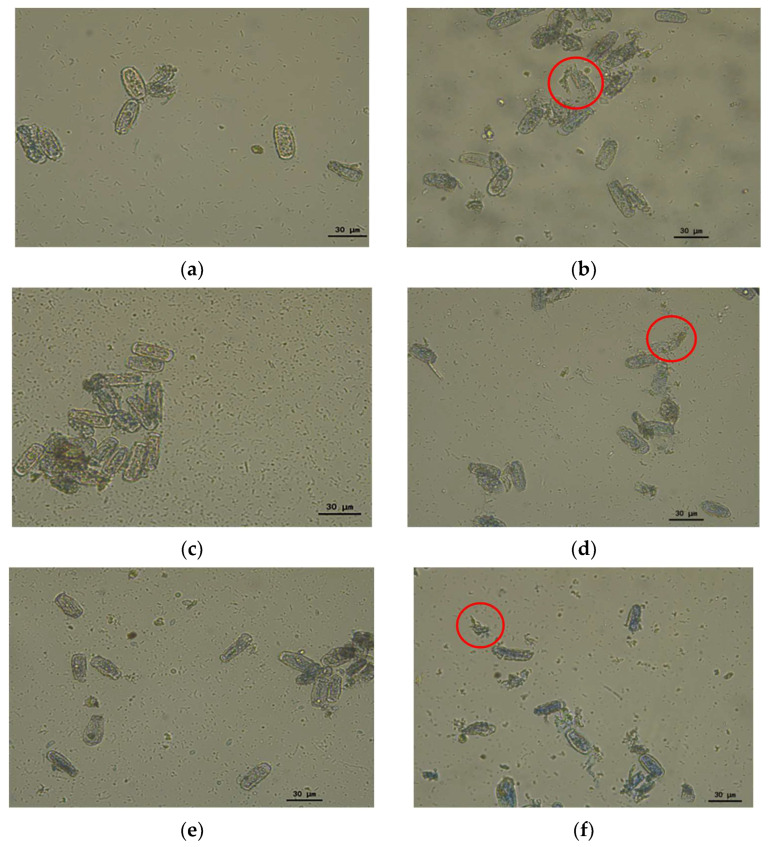
(**a**) powdery mildew spores treated with sterile water for 1 day; (**b**) powdery mildew spores treated with enzyme for 1 day; (**c**) powdery mildew spores treated with sterile water for 2 days; (**d**) powdery mildew spores treated with enzyme for 2 days; (**e**) powdery mildew spores treated with sterile water for 4 days; and (**f**) powdery mildew spores treated with enzyme for 4 days. Red circles indicate spores with different degrees of fragmentation. Scale bar = 30 μm.

As shown in Figure 11, the disruptive effect on spores of *P. pannosa* was clearly observed following enzyme treatment. In the control group, most powdery mildew spores exhibited intact cell walls, full intracellular contents, and strong viability. After 1 day of enzyme treatment at 25 °C, one or two spores showed initial cell wall disruption, and a small proportion of spores were stained by trypan blue, indicating loss of viability. By day 2, the number of spores with disrupted cell walls had increased significantly. After 4 days of treatment, most spores exhibited severe cell wall rupture accompanied by obvious leakage of intracellular contents, and the majority of spores were stained by trypan blue, indicating cell death. These results demonstrated that the enzyme exerted detectable effects on powdery mildew spores as early as the first day of treatment, with pronounced antifungal activity observed by day 4. The Aag25 protein was able to disrupt the spore cell wall and induce leakage of intracellular contents in powdery mildew spores. This phenomenon was consistent with our previous observations obtained when strain KMR13 was re-inoculated onto powdery mildew spore masses.

## 4. Discussion

Current studies have shown that microorganisms capable of producing β-1,3-glucanases mainly include bacteria and fungi, while some plants also possess this ability. In bacteria, reported producers include *Paenibacillus polymyxa* [28], *Streptomyces* sp. [29], *Pyrococcus furiosus* [30], and *Bacillus lehensis* G1 [31]. In fungi, β-1,3-glucanase production has been reported in *Phanerochaete chrysosporium* [32], *Trichoderma asperellum* [33], and *Aspergillus fumigatus* [34]. In plants, sugarcane [35] and grapevine [36] have also been reported to produce this enzyme. At present, most studies have focused on changes in β-1,3-glucanase activity during plant infection and on the research and application of transgenic plants. For example, in vitro antifungal assays conducted by Zhang et al. [37] demonstrated that β-1,3-glucanase exerted significant inhibitory effects against common fungal pathogens of wheat grains, including *Alternaria nigra*, *Alternaria alternata*, and *Alternaria flavus*. The inhibitory effects were mainly reflected in interference with spore germination and hyphal morphogenesis. Taif et al. [38] introduced and overexpressed the β-1,3-glucanase gene *PnGlu1* in tobacco, and the transgenic tobacco plants exhibited enhanced resistance to infection by *Fusarium solani*. Studies on β-1,3-glucanases in fungi, particularly fungi with antifungal activity, have thus far concentrated mainly on the genus *Trichoderma* [39,40], whereas related studies on fungi of the genus *Alternaria* remain to be further explored.

Currently, the primary host organisms used for the recombinant expression and production of β-1,3-glucanases include *Escherichia coli*, *Pichia pastoris*, and *Bacillus* species. This study selected the *E. coli* expression system, which is more technically mature and relatively simpler to operate compared with yeast expression systems. It is widely used in the discovery and production of novel β-1,3-glucanases, such as those from *Pichia guilliermondii* [41], the moose rumen microbiome metagenome [42], and *Pseudomonas aeruginosa* [43]. However, the *E. coli* expression system also has some drawbacks, such as the lack of post-translational modification capabilities. For proteins that require post-translational modification to achieve full biological activity, it is preferable to select eukaryotic cells as the host. Additionally, fusion proteins expressed in *E. coli* often appear as inclusions and must undergo renaturation and refolding to become active proteins, which complicates the purification process and results in significant loss of the target protein. Nevertheless, compared with other expression systems, *E. coli* offers a well-characterized genetic background, ease of use, rapid growth suitable for large-scale fermentation, strong tolerance for many proteins, and the ability to express these proteins at high levels. Consequently, it remains the most widely used expression system at present [44].

In our previous study, a complementation assay was performed with the mycoparasitic strain KMR13. The results indicated that when KMR13 was reintroduced to powdery mildew conidia, it caused conidial cell wall disruption and leakage of intracellular contents. In the present study, treatment of rose powdery mildew conidia with β-1,3-glucanase induced similar phenomena. Li et al. [45] cloned and expressed the chitinase gene *CHI10* from *Trichoderma viride* and found that *CHI10* could compromise rust spore cell walls. Li Mingjiao [46] reported that recombinant chitinase *CaChi93* caused thinning of the rust spore cell wall and subsequent rupture, leading to leakage of intracellular contents. Sui Wenjing [47] demonstrated that recombinant β-1,3-glucanase *PKG1* could kill rust spores; throughout the process, the cell wall remained morphologically intact, but notable denaturation and leakage of intracellular contents occurred. These findings indicate that chitinases *CaChi93* and *CHI10* can directly disrupt rust spore cell wall integrity, causing wall thinning, wrinkling, and rupture, consistent with their enzymatic function in degrading chitin scaffolds.

In contrast, β-1,3-glucanase *PKG1* also induced rust spore death, but the cell wall remained structurally intact, undergoing only denaturation and intracellular leakage. This suggests that *PKG1* does not fully degrade the cell wall but rather modifies or hydrolyzes its glucan components, altering the wall structure and permeability and thereby affecting spore viability and membrane integrity. Although both are β-1,3-glucanases, *PKG1* preserved the rust spore cell wall integrity, whereas in the present study, *Aag25* caused powdery mildew conidial wall rupture and cytoplasmic leakage. This discrepancy likely reflects differences in the composition, crosslinking, and structural stability of rust and *P. pannosa* spore walls. Powdery mildew conidia may contain more exposed or easily degradable β-1,3-glucan or lack sufficient reinforcing components (e.g., proteins), making them more sensitive to glucanases. In subsequent applied research, enzyme formulations can be selected or designed based on the fine structure of the target pathogen’s spore wall to optimize substrate specificity and efficacy. Collectively, these studies demonstrate that individual chitinases or glucanases can damage spores under different conditions, but the extent and manifestation of damage vary. Chitin and glucans are the main structural polysaccharides in fungal cell walls and often crosslink to form a robust network. Selim et al. [48] showed that chitinases and β-1,3-glucanases exhibit effective inhibition against postharvest fungal pathogens on tomato fruits. Systems co-expressing both enzymes displayed stronger antifungal activity than single-enzyme treatments, likely due to the synergistic disruption of the polysaccharide network. This enzyme-based composite biocontrol strategy shows potential advantages over conventional chemical fungicides in agricultural disease management. Therefore, future studies should evaluate the synergistic effects of enzyme combinations to inform the development of highly effective composite enzyme formulations.

In this study, the *Aag25* gene was heterologously expressed in a prokaryotic system, and its enzymatic activity was characterized. Future work may focus on a detailed investigation of its enzymatic properties, optimizing the expression conditions of the β-1,3-glucanase to enhance both yield and activity. Additionally, the *Aag25* gene could be expressed in a eukaryotic system to compare the activity of recombinant proteins produced in prokaryotic versus eukaryotic hosts. There are abundant gene resources in the genome of strain KMR13, in which chitinase genes belonging to GH18 family account for a considerable proportion. Specific chitinase genes that can play a synergistic effect with Aag25 protein can be screened from these genes, and further cloning and expression research can be carried out. A comprehensive analysis of the interaction mechanism between these enzymes will not only elucidate their functional complementarity but also provide important insights into the role of cell wall-degrading enzymes during the mycoparasitic process.

## 5. Conclusions

In conclusion, 30 β-1,3-glucanase genes were identified in the genome of *Alternaria alternata* KMR13, and transcriptome analysis showed that 23 of these genes were expressed after induction with *P. pannosa* spores from Chinese rose, among which 7 were continuously upregulated during the 0–96 h period. RT-qPCR further confirmed that the selected genes were significantly induced, with peak expression at 24 h. These results suggest that KMR13 may exert its biocontrol activity by secreting β-1,3-glucanases and other cell wall-degrading enzymes to damage the cell wall of the pathogen and cause leakage of intracellular contents. Among the candidate genes, the GH17 family β-1,3-glucanase gene *Aag25* was selected for functional analysis, and the recombinant Aag25 protein was successfully expressed and purified. Enzyme activity assays showed that recombinant Aag25 markedly disrupted the cell walls of *P. pannosa* spores from Chinese rose, leading to cytoplasmic leakage and indicating that *Aag25* is likely involved in cell wall degradation during KMR13 mycoparasitism, playing an important role in its biocontrol mechanism.

## Figures and Tables

**Table 1:** RT-qPCR primers.

Primer Name	Primer Sequences (5′–3′)
Actin-F	ATGTGCAAGGCCGGTTTCG
Actin-R	GCGAGCTGTCAGAAAGTGCT
Aag2-F	GTGGTGTTGGTGGAGAGTGG
Aag2-R	TGCTCGTCATAGCACCAGAG
Aag3-F	TGGGGAGCTTATTGGGTGAG
Aag3-R	TGGAAGAATAACGAAGGATAAGCC
Aag5-F	GGCCAAGCGTTAGATACGCA
Aag5-R	TGTACCGTTTTGTGCCATCCA
Aag10-F	TTTGCCGTGCGTGATTTCTG
Aag10-R	CAGACGTGGGTGAGTCTAGC
Aag22-F	GATAGCGAAGGTGGCTTGGA
Aag22-R	AACATACTGAGCACACGCCA
Aag25-F	AATGGAAAGGCTGCTGACGA
Aag25-R	GTCCCCCGTCGTCTATGTTC

**Table 2:** Standard dilution table.

Pipe Number	Concentration Before Dilution (mg/mL)	Volume of the Standard (μL)	Volume of Distilled Water (μL)	Concentration After Dilution (mg/mL)
1	10	100	900	1
2	1	160	40	0.8
3	1	120	80	0.6
4	1	80	120	0.4
5	1	40	160	0.2
6	1	20	180	0.1
7	1	10	190	0.05

**Table 3:** Sample determination.

Reagent Name (μL)	Test Tube	Control Tube	Standard Tube	Blank Tube
Sample	35	-	-	-
Inactivated sample	-	35	-	-
Standard	-	-	35	-
Distilled water	-	-	-	35
Reagent 1	35	35	35	35
Mixed thoroughly and placed in a 37 °C water bath for 60 min				
Reagent 2	230	230	230	230

**Table 4:** Basic physical and chemical properties of β-1,3-glucanase gene in KMR13.

Gene ID	Number of Amino Acids	Molecular Weight (Da)	pI	Signal Peptide	Subcellular Localization	Transmembrane Domains	Glycosidase Family
*Aag1*	668	72,260.13	5.34	−	cyto	0	GH16
*Aag2*	379	38,852.28	4.37	+	extr	0	GH16
*Aag3*	443	46,808.19	4.58	+	extr	0	GH16
*Aag4*	442	46,888.91	4.73	+	extr	0	GH16
*Aag5*	500	51,480.46	4.33	+	extr	0	GH16
*Aag6*	375	41,264.72	4.60	+	extr	0	GH16
*Aag7*	354	37,974.11	5.81	−	extr	0	GH16
*Aag8*	580	62,205.78	5.21	−	extr	0	GH16
*Aag9*	605	62,811.82	4.46	+	extr	0	GH16
*Aag10*	479	54,501.79	5.85	−	plas	1	GH16
*Aag11*	1807	203,696.69	7.33	−	pero	1	GH16
*Aag12*	780	86,910.64	5.34	−	plas	1	GH16
*Aag13*	1082	120,906.48	6.43	+	plas	10	GH16
*Aag14*	448	50,524.92	8.75	−	pero	1	GH16
*Aag15*	412	46,051.07	6.39	−	mito	0	GH16
*Aag16*	661	73,694.75	4.66	−	plas	1	GH16
*Aag17*	352	38,425.02	4.43	+	extr	0	GH16
*Aag18*	382	42,885.52	4.45	+	extr	0	GH16
*Aag19*	423	44,800.07	4.74	+	extr	0	GH17
*Aag20*	667	72,346.18	4.93	−	plas	1	GH17
*Aag21*	643	68,279.16	5.41	+	extr	0	GH17
*Aag22*	620	65,105.66	4.77	+	mito	0	GH17
*Aag23*	380	40,367.16	6.10	+	extr	0	GH17
*Aag24*	270	29,172.91	4.20	+	extr	0	GH17
*Aag25*	305	32,685.72	5.21	+	extr	0	GH17
*Aag26*	310	33,378.99	4.97	+	extr	0	GH17
*Aag27*	413	45,142.70	5.76	−	extr	0	GH64
*Aag28*	436	47,301.93	8.84	−	mito	0	GH64
*Aag29*	727	81,018.06	6.41	−	mito	0	GH81
*Aag30*	968	106,539.41	5.90	−	extr	0	GH81

mito = mitochondria; plas = plasma membrane; cyto = cytoplasm; extr = extracellular space; pero = peroxisomes.

## Data Availability

The sequencing data presented in this study are openly available from the National Center for Biotechnology Information (NCBI) repository under BioProject: PRJNA1186384.
